# Fludarabine, a Potential DNA-Dependent RNA Polymerase Inhibitor, as a Prospective Drug against Monkeypox Virus: A Computational Approach

**DOI:** 10.3390/ph15091129

**Published:** 2022-09-09

**Authors:** Hisham N. Altayb

**Affiliations:** 1 Department of Biochemistry, Faculty of Sciences, King Abdulaziz University, Jeddah 21589, Saudi Arabia; hdemmahom@kau.edu.sa; 2 Centre for Artificial Intelligence in Precision Medicine, King Abdulaziz University, Jeddah 21589, Saudi Arabia

**Keywords:** MPXV, antiviral, poxviruses, RNA polymerase, glycoprotein

## Abstract

Monkeypox is a zoonotic contagious disease that has recently re-emerged in different countries worldwide. Due to the lack of an effective treatment that eliminates the virus, there is an urgent need to find effective drugs to stop the spread of the multi-country outbreak. The current study aimed to use computational methods to quickly identify potentially effective drug*s* against the *Monkeypox* virus (MPXV). Three MPXV proteins were targeted in this study due to their essential role in viral replication (a DNA-Dependent RNA Polymerase subunit (A6R)), a protein involved in cell entry (D8L), and a protein catalyzing the envelopment of intracellular mature virus particles (F13L). We virtually screened a library of 1615 FDA-approved compounds, utilizing different in-silico approaches including computational modeling, molecular docking, molecular dynamic (MD) simulation, and MM-GBSA. The compound Fludarabine was found to have the best docking score (−7.53 kcal/mol) in relation to the MPXV A6R protein. Additionally, Fludarabine showed in-silico activity on the D8L and F13L proteins. During the whole period of the 100 ns MD simulation, the complex of A6R and Fludarabine exhibited the best stability. This stability was reflected in a good score of MM-GBSA, with an average value of −44.62 kcal/mole in a range between −53.26 and −35.49 and a low value of standard deviation (3.76). Furthermore, Fludarabine blocked efficiently the Asn175 residue which has an important role in the attachment of the virus to a host cell. The results of this study recommend more in vitro studies on this compound, as a starting point to develop a novel treatment against MPXV.

## 1. Introduction

Monkeypox is a zoonotic disease caused by the Monkeypox virus (MPXV) which belongs to the Orthopoxvirus genus of the Poxviridae family. This family contains other human-associated pathogens, including the vaccinia (VACV), cowpox (CPXV), and variola (VARV) viruses [1]. The virus circulates among a number of mammals, with human transmission on rare occasions [2]. The virus was first identified in cynomolgus monkeys in a laboratory in Denmark in 1958 [3]. The first human case was reported in the Republic of Congo in 1970, infecting a neonate aged 9 months [3]. Since that time, different outbreaks have been reported sporadically in different countries including South Sudan (2005) [4], Congo (2009), and Central African Republic (2016), and two outbreaks were reported in Nigeria, the first one between 1971 and 1978, and the last one on 26 September 2017 [5]. Outside of the African countries, an outbreak occurred in the United States in 2003 [6]. Recently, as of 25 May 2022, a total of 195 confirmed cases have been documented in non-endemic European countries including Austria (1), Belgium (4), Czech Republic (1), Denmark (1), France (5), Germany (5), Italy (5), The Netherlands (6), Portugal (37), Slovenia (1), Spain (51), Sweden (1) [2], and the UK (77) [7].

The draft genome [8] and complete genomes of the newly emerged MPXV have been released recently; the complete genome was obtained from a confirmed MPXV case in Massachusetts, United States (ON563414.3), and consists of 197,205 bp nucleotide sequences [9]. All cases reported up to 25 May 2022 showed high similarity to the West African clade [10]. MPXV and variola are closely related genetically and serologically. The central region of the variola virus showed 96.3% similarity to the MPXV sequence; this region contains information for the most essential enzymes and structural proteins. In contrast, the end regions of these genomes present few differences and contain virulence and host-range genes [11].

**Figure 1:**
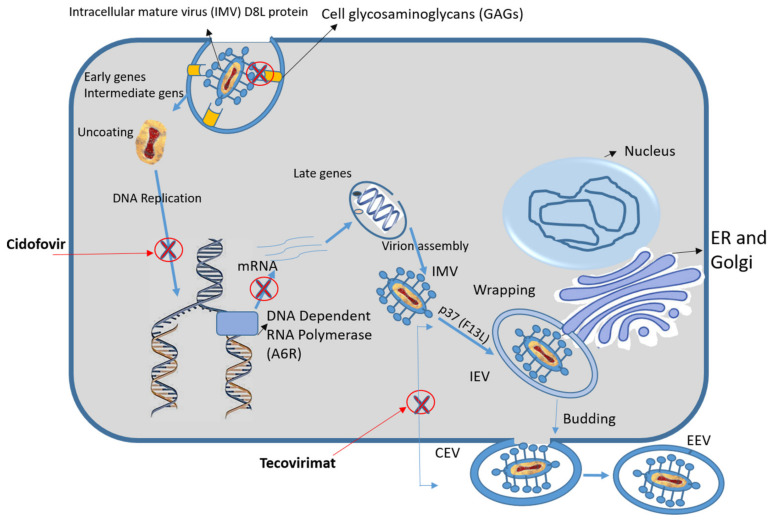
Schematic representation of the MPXV replication cycle, showing the protein targets of antiviral agents (Cidofovir and Tecovirimat) and the studied compound (Fludarabine); the antiviral targets are indicated by red crosses.

Transmission of the MPXV from an infected animal to humans can occur via an animal bite or scratch or through direct contact with an infected animal’s blood, body fluids, meat, or lesions [2]. The virus uncoating and production of early genes starts right away upon the attachment and fusion of the virus with the host cell. DNA replication starts at this time, followed by the transcription of intermediate and late genes [12]. With the help of the F13L protein (also known as p37), the intracellular mature virions (IMVs) are assembled and wrapped by membranes of the endoplasmic reticulum (ER) or Golgi apparatus and could remain in the host cells as cell-associated virus (CEV) or migrate outside the cells as extracellular enveloped virus (EEV) particles [13] (Figure 1).

Several methods have been used in the treatment of Orthopoxvirus infections, including siRNA-mediated replication inhibition via targeting the VACV genes (B1R, G7L, and D5R) and MKPV genes (D8L and A6R) [14]. Different MPXV proteins were targeted due to their essential role in viral replication (RNA polymerase, A6R), cell entry (GAG-binding IMV membrane protein, D8L), and the catalysis of the envelopment of intracellular mature virus particles (palmitoylated extracellular enveloped virus (EEV) membrane glycoprotein coded by the F13L gene) [14,15].

A new synthesized antiviral drug called ST-246 (Tecovirimat) is being designed to treat human infections with Orthopoxviruses. The activity of Tecovirimat (ST-246) on the vaccinia and cowpox viruses was discovered as a result of the high-throughput screening of 356,240 compounds. Tecovirimat works by inhibiting the conserved protein (F13L) in Orthopoxviruses, preventing the formation of EEV [16]. Tecovirimat demonstrated the best in vitro activity in Vero cell cultures and demonstrated a good cellular toxicity profile, with low 50% cytotoxic concentration (CC_50_) values in different cell lines including human, mouse, rabbit, and monkey cells. In addition, the compound reduced (30–40%) the growth rate of cells compared to non-treated cells [17]. Cidofovir is currently approved as an antiviral agent against Orthopoxviruses and Cytomegalovirus (CMV). It is a nucleotide analogue that inhibits the viral DNA polymerase after being converted to phosphorylated Cidofovir by cellular kinases [12,18]. Renal toxicity is one of the known side effects of Cidofovir [12].

Fludarabine is a known anticancer agent that was reported to have inhibitory activity on ZIKV, SFTS Phlebovirus, and Enterovirus A71 [19]. Fludarabine phosphate is a chemically synthesized nucleotide antimetabolite analog of vidarabine (ara-A), which is dephosphorylated to F-ara-A to enter the cells and then is converted into the active triphosphate form, resulting in the inhibition of the process of DNA replication via the inhibition of a ribonucleotide reductase, a DNA polymerase, a DNA primase, and a ligase. Additionally, Fludarabine triphosphate could be incorporated into RNA resulting in the blocking of the transcription process [20,21]. In this study, Fludarabine exhibited excellent docking scores, MD simulation, and MM-GBSA profile in relation to different MPXV proteins.

Several types of anti-MPXV agents have been studied, including Tecovirimat (ST-246), Cidofovir, and Brincidofovir.

Unfortunately, a successful treatment for human infections has not yet been found [15], and up to August 2022, there is no available treatment for MPXV infection [22]. In the absence of a specific therapy, there is an urgent need to discover new effective and safe treatments to monitor the current outbreak. Here, we targeted several MPXV proteins using different in-silico tools to rapidly identify a possible antiviral agent among drugs already approved by The United States Food and Drug Administration (USFDA or FDA).

## 2. Results and Discussion

### 2.1. Sequence Alignment

The protein sequences of the newly emerged MPXV (URK20553) were compared to those of different Monkeypox viruses; high similarity was found, especially in the binding site, as shown in Figure S1. As presented in our study, the A6L, D8L, and F13L proteins of the newly emerged virus appeared to be highly conserved among MPXV, in agreement with previous studies [23,24].

### 2.2. Modeling and Active Site Prediction

**Figure 2:**
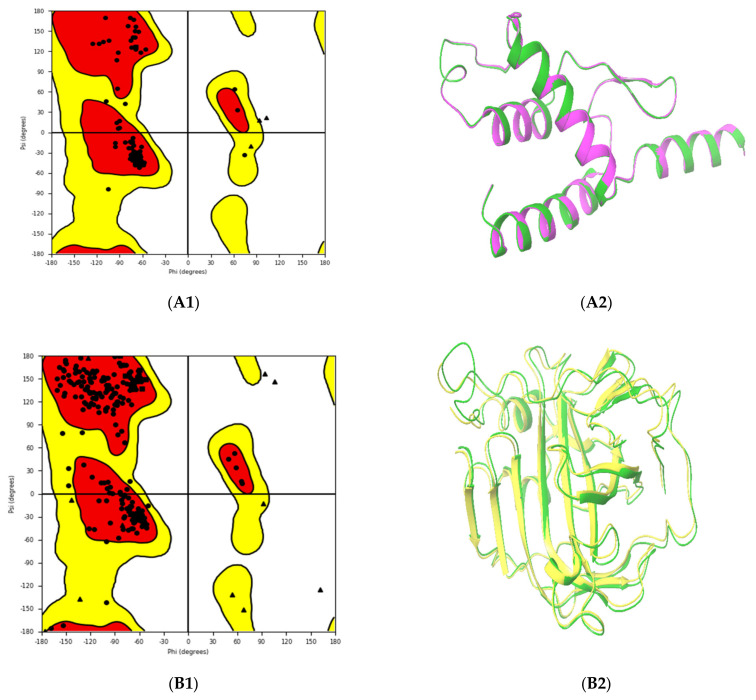

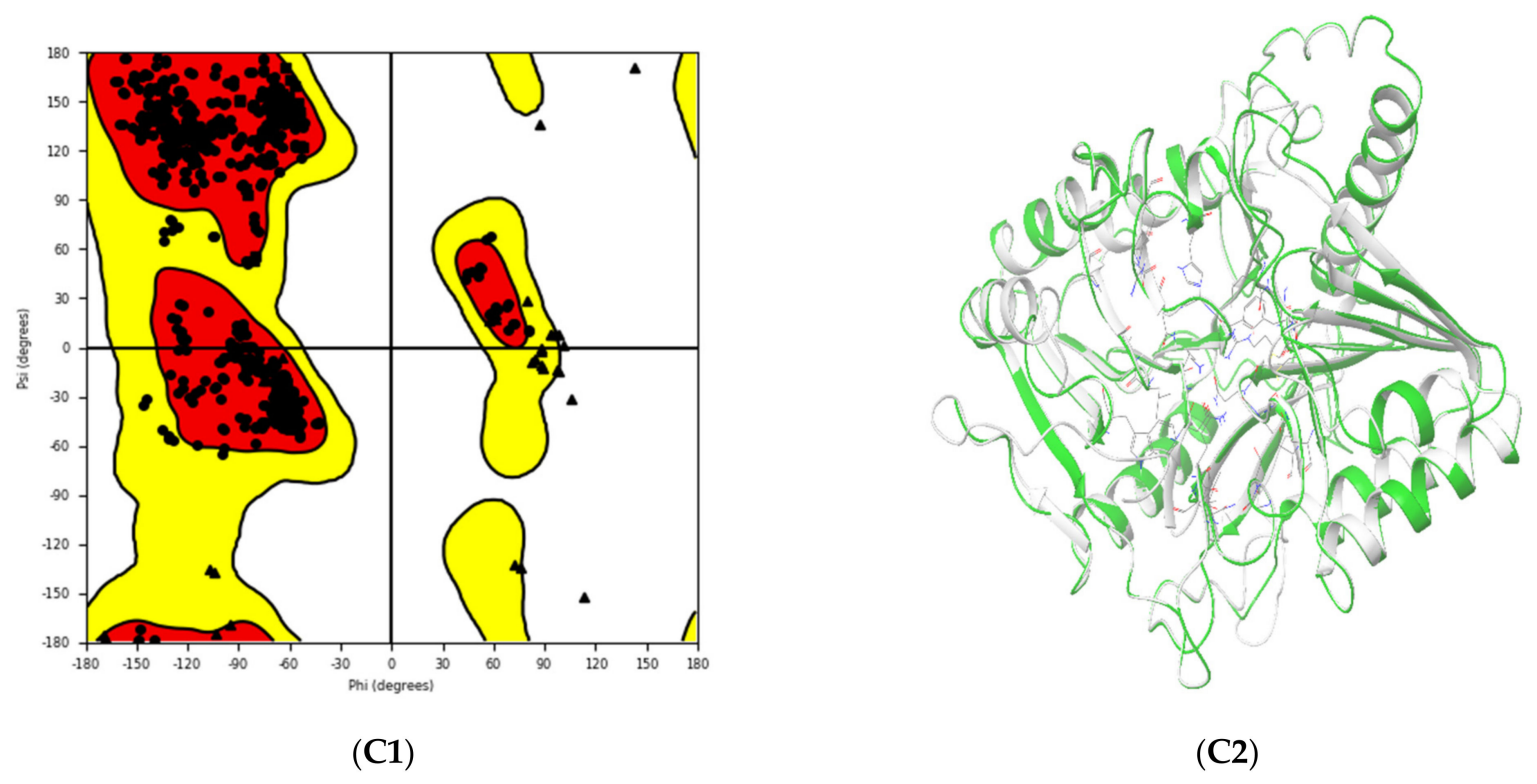
Ramachandran plots of the generated protein structures (Left). The red colors in the Ramachandran plots indicate the most allowed regions; the generously allowed regions are shown in yellow, and the disallowed regions are shown in white. The triangles indicate glycine, the squares indicate proline, and all other residues are plotted as circles. On the right of the figure, the superimposition of the generated structures in 3D (green) with those of reference proteins that were used as a template for the model generation is shown; their PDB IDs are: 6RFL_C (violet), 4E9O_A (yellow), and 7E0M_A (white). (**A**). DNA-Dependent RNA Polymerase subunit (A6R), (**B**). Carbonic anhydrase (GAG-binding IMV membrane protein) (D8L), (**C**). Palmitoylated extracellular enveloped virus (EEV) membrane glycoprotein (F13L).

**Figure 3:**
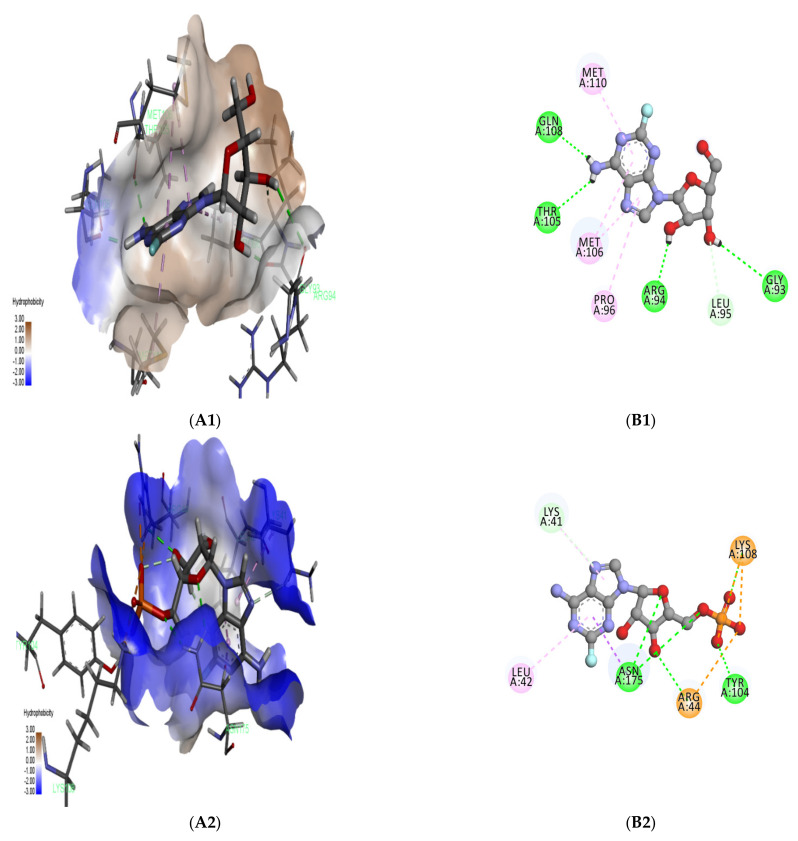

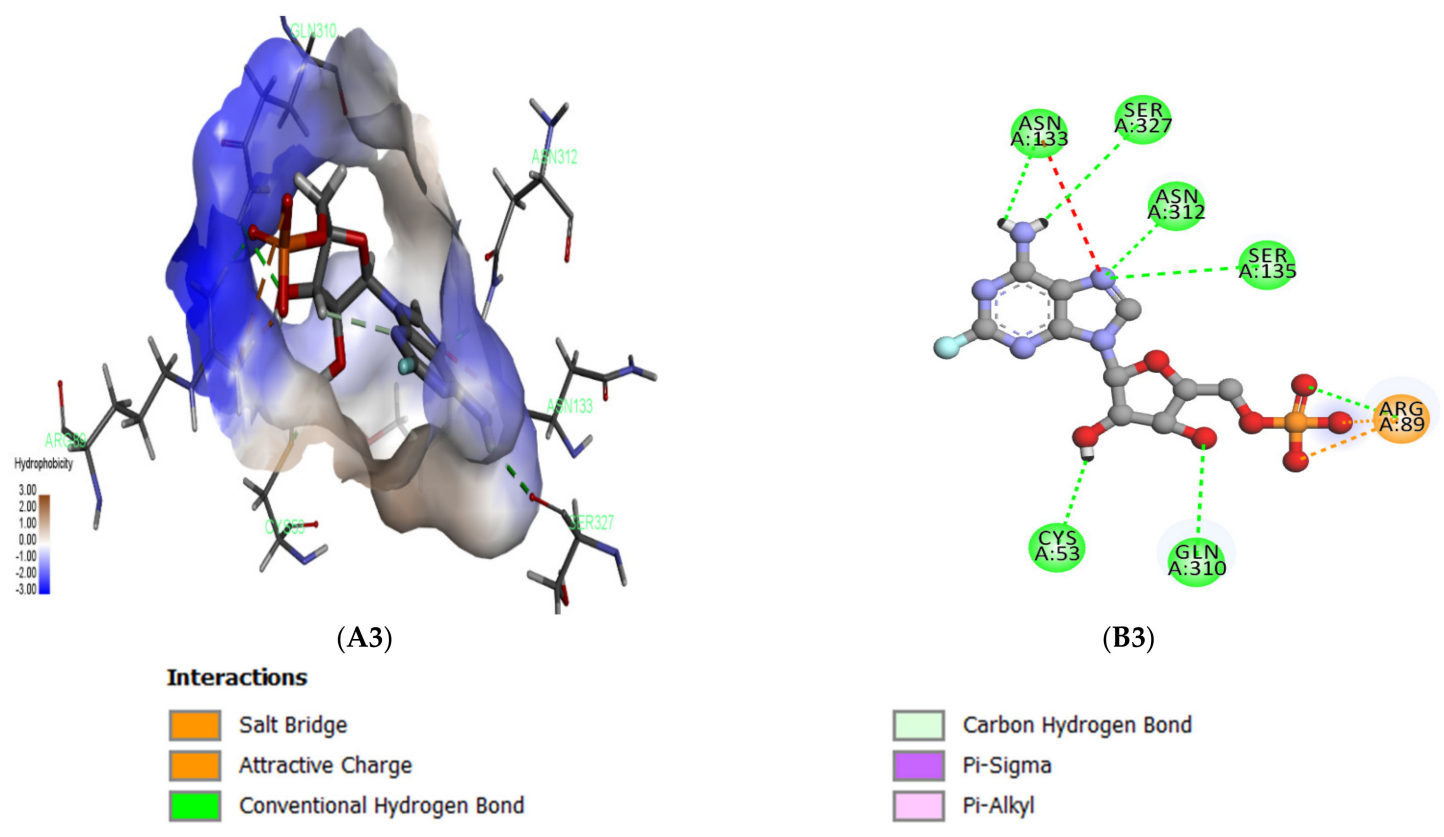
Image of 3D (**A**) and 2D (**B**) interactions of ligands and proteins during the XP docking process. The 3D structures show the binding pocket of the proteins including the positioning of the ligands; the binding pocket colors in the 3D plots correspond to residues’ hydrophobicity, while the colors in the 2D plots correspond to different types of bonds. (**A1**,**B1**) complex of A6R and Fludarabine, (**A2**,**B2**) complex of D8L and Fludarabine, (**A3**,**B3**) complex of F13L and Fludarabine.

Three-dimensional (3D) structures of the selected proteins were generated using the Prime tool. The generated 3D structures were validated using the Ramachandran plot, which showed that 99% of A6R and D8L residues were located in their highly preferred regions, and only 1% of these residues were located in their preferred region. The generated structure of F13L showed that 94.4% of its residues were located in the highly preferred regions, 4.1% of the residues were located in the preferred regions, and only 1.5% of the residues were located in the permitted region (Figure 2A1,C1 and Figure 3B1). The comparison of the generated structure with references used for the building of the structures indicated high similarity especially at the binding sites, as shown in Figure 2A2,C2, and Figure 3B2. In the protein sequence alignment, the active site residues were highly similar and conserved among different poxviruses, which supported the usage of other poxviruses protein models as a template for 3D structure generation [25]. Furthermore, the RMSF was used to study the stability of the generated proteins. During the MD simulation, the C-alphas, backbones residues, and heavy atoms of the A6R structure were highly stable. A small fluctuation was observed at the beginning, and 84% (16/19) of the interacting residues were located in a highly stable region (Figure 6A). Though the structures of D8L and F13L were stable during a 100 ns MD simulation, relatively larger fluctuations were observed between residues 204 and 213 in D8L and between residues 300 and 312 in F13L, which correspond to loops. Interestingly, as shown in Figure 6B,C, 90% (27/30) of D8L interacting residues and 95% (37/39) of F13L interacting residues appeared located in a highly stable region.

Active site prediction was achieved via the Maestro SiteMap tool. The active site of A6R protein consists of Leu90, Pro96, Thr105, Met106, Gln108, Met110, and Val113. The A6R binding site scored 0.7 for both Dscore and SiteScore and is considered an intermediate druggable binding site. The prediction of the D8L binding site revealed the presence of the following residues: Gly40, Lys41, Leu42, Arg44, Ser64, Thr65, His67, Tyr69, Val94, Tyr104, Lys108, Ile116, Thr173, Ile174, Asn175, Ser177, Trp182, Ser204, Ser205, Asn207, His208, His213, Tyr214, Ile215, Thr216, Glu217, Asn218, Tyr219, Arg220, and Asn221. The active site of the D8L protein contains a residue (Asn175) that has an important role in host cell binding [23], which supports our finding. The D8L binding site scored 0.97 for Dscore and 1.02 for SiteScore and is considered a very druggable binding site. The binding site of F13L consists of Phe52, Cys53, Asn55, Gly87, Leu118, Gly119, Cys120, Asn133, Ala134, Thr137, Gly139, Ser140, Ile144, Leu239, Ala240, Val242, Arg246, Trp279, Gln310, Asn311, Asn312, Ala328, Asn329, Asp331, His334, and Leu339. It scored 0.93 for Dscore and 1 for SiteScore and is considered a very druggable binding site [26].

### 2.3. Molecular Docking

**Figure 4:**
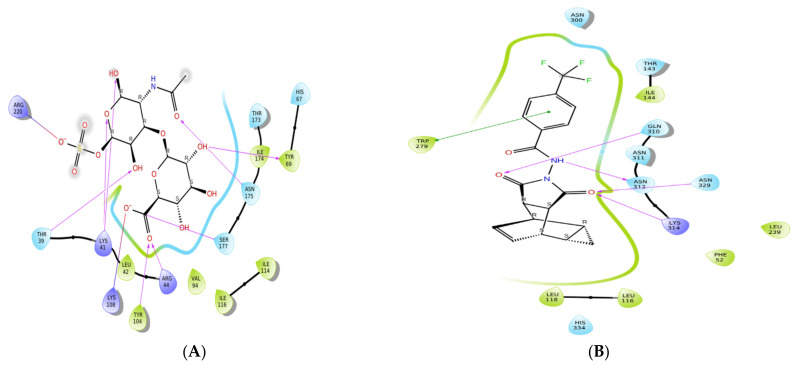

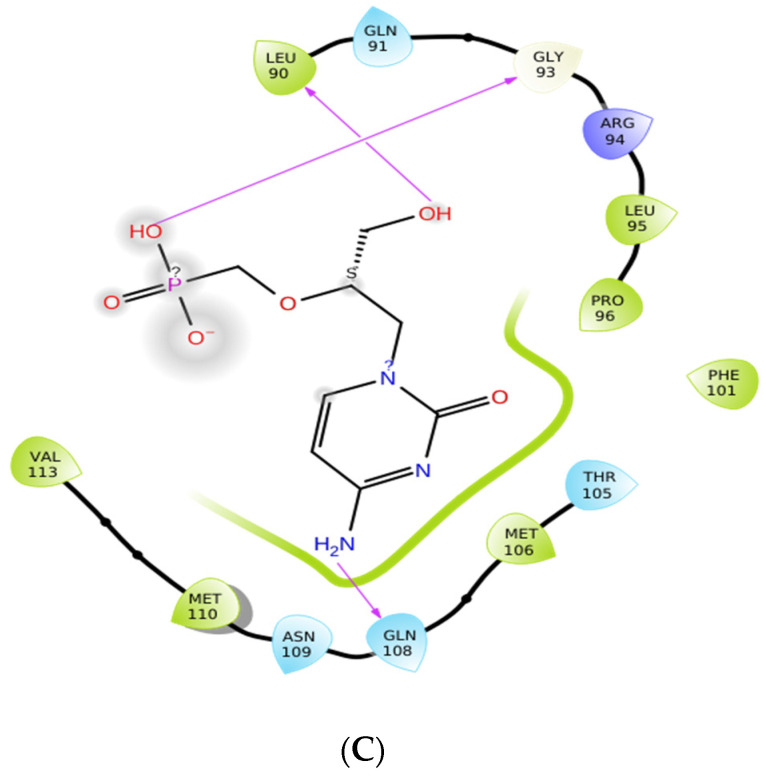
Image of the 2D interaction of the control compounds used for the comparison of the docking results, and proteins. (**A**) Chondroitin sulfate and D8L, (**B**) Tecovirimat and F13L, (**C**) Cidofovir and A6R.

Three essential MPXV proteins were used as targets for virtual screening. These proteins are essential for host cell entry (D8L), catalysis of the envelopment of intracellular mature virus particles (F13L), and viral replication (A6R). Different previous studies used these proteins for the development of anti-MPXV agents [1,15,23]. The strategy of blocking viral cell entry has been explored as a successful antiviral procedure for Poxviruses [23]. In this study, we used molecular docking to study the potential activity of FDA-approved drugs against MPXV. Among the screened compounds, Fludarabine exhibited the best docking score (−7.53 kcal/mol) in relation to the A6R protein and exhibited good docking scores in relation to the D8L (−6.64 kcal/mol) and F13L (−7.66 kcal/mol) proteins (Figure 3A1,B1) (Table 1). Four stable H-bonds (involving Gln108, Thr105, Arg94, and Gly93 residues) were associated with the interaction between Fludarabine and A6R. Interestingly, Fludarabine is a known anticancer agent that was reported to have inhibitory activity on ZIKV, SFTS Phlebovirus, and Enterovirus A71 [19], in particular, anti-replication activity. This is in agreement with our study. The complex of D8L and Fludarabine exhibited two hydrogen bonds (Asn175, and Tyr104), two salt bridges (Lys108 and Arg44), one carbon–hydrogen bond (Lys41), and one pi-alkyl bond (Leu42) (Figure 3A2,B2). The blocking of Ile174 with a strong (with a short distance) H-bond could interfere with the interaction with the D8L protein, due to the essential role of the Asn175 residue in host cell binding [23]. As observed in Figure 4A, chondroitin sulfate, which mediates the binding of D8L to the host cell [27], showed an interaction with Asn175, Tyr104, Lys108, Arg44, Lys41, and Leu42, similar to Fludarabine. This indicates the possibility of a competitive inhibitory activity of Fludarabine. The complex of F13L and Fludarabine exhibited seven hydrogen bonds (involving Cys53, Arg89, Asn133, Ser135, Gln310, Asn312, and Ser327) and one salt bridge (involving Arg89). Fludarabine and Tecovirimat (the control) exhibited a similar interaction with F13L; they showed similar interacting residues (Gln310 and Asn312) (Figure 3A3,B3).

### 2.4. Molecular Dynamic Simulation

MD simulation is used to study the conformational changes of ligands in the binding pocket of a protein [28]. In this study, the stability and dynamic behavior of docked complexes were evaluated using MD simulation. The RMSD values of the complexes (Fludarabine with A6R, D8L, and F13L proteins) were investigated during 100ns of simulation. In general, the RMSD values of all three complexes were less than 3Å, which indicated the stability of bound ligands in the protein binding grooves [29].

**Figure 5:**
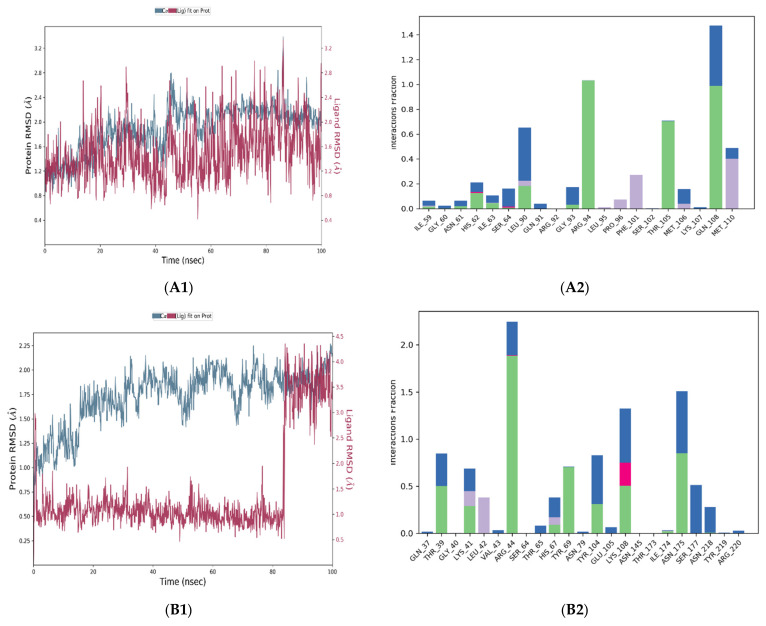

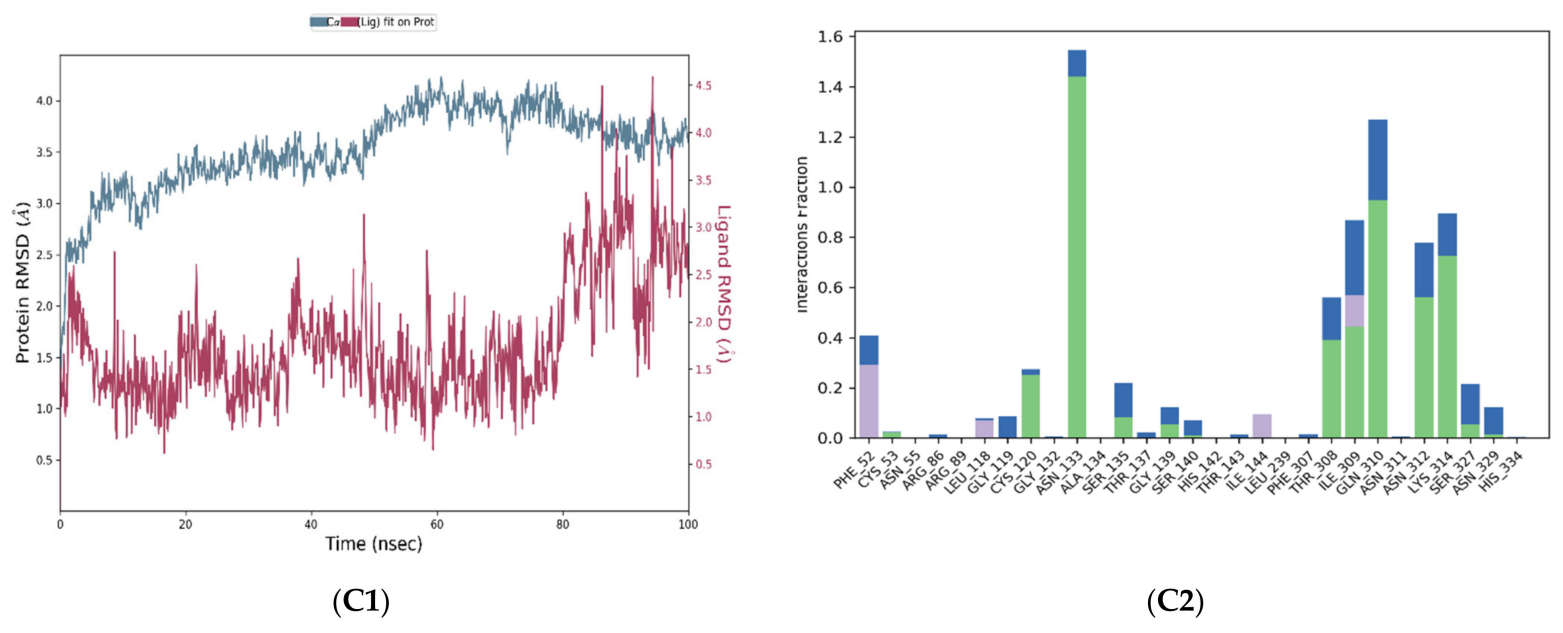
RMSD plot (**left**) and histogram (**right**) describing the interaction of ligands and proteins during the 100 ns simulation. The RMSD plot shows ligands in red and protein C-alpha atoms in blue; the histogram shows H-bonds (green), water bridges (blue), and hydrophobic interactions (purple). (**A**). Complex of A6R and Fludarabine, (**B**). Complex of D8L and Fludarabine, (**C**). Complex of F13L and Fludarabine.

As can be observed in Figure 5A1, Fludarabine (red) aligned with the A6R protein backbones (blue) from the beginning of the simulation until the end. The ligand showed a small acceptable fluctuation (2.8 Å) and remained aligned with the protein backbones from the beginning of the simulation until the end. This good stability was supported by the presence of three stable (that existed in more than 70% of the simulation time) hydrogen bonds (involving Arg94, Thr105, and Gln108); another stable water bridge was observed with Gln108 (Figure 5A2). These results are aligned with the docking result that showed two stable H-bonds with Arg94, Thr105, and Gln108. The docking complex of Fludarabine and A6R showed better stability than the complex of Cidofovir and A6R, as shown in Figure S2.

The RMSD plot of the D8L and Fludarabine complex is shown in Figure 5B1. In this complex, the ligand showed lower stability (fluctuating in the range between 0.4 and 2.2 Å) during the MD simulation, but still acceptable (less than 3 Å). The ligand separated from the protein backbone atoms from the beginning of the simulation, then, after 85 ns, it remained aligned with the protein backbones until the end. A very stable H-bond (observed in more than 100% of the simulation time) occurred with Arg44 and Asn175. These residues have an important role in the binding of chondroitin sulfate which mediates the cell binding [23]; three additional water bridges were observed with Arg44, Tyr104, Lys108, Asn175, and Ser177 (Figure 5B2). The interaction of chondroitin sulfate and D8L showed an interaction with Arg44 and Asn175 in less than 80% of the simulation time (Figure S3B), which supports the possibility that Fludarabine exerts a competitive inhibitory activity. 

F13L is an essential viral protein required for the catalysis of the envelopment of intracellular mature virus particles [15]. In this study, Fludarabine and F13L showed a stable interaction during 100 ns of simulation, and the ligand deviated from the protein backbones during the whole simulation time, in the range between 0.7 and 3.5 Å; however, the ligand’s stability remained in an acceptable range (Figure 5C1). Three stable H-bonds were observed with Asn123, Gln310, and Lys314. Additionally, two stable water bridges (which occurred with Ile309 and Gln310) formed as a result of the interaction of F13L and Fludarabine (Figure 5C2). As shown in Figure S4A, the RMSD of F13L and Tecovirimat was similar to that of F13L and Fludarabine; in each of them, the ligands were separated from the protein backbone from the start to the end of the simulation.

Furthermore, as shown in Figure 7, we calculated the rGyr to estimate the ligand extension, the MolSA to estimate the molecular surface area, the solvent-like behavior of the complex via SASA, and the ligand polar area via PSA [30]. Usually, the lower the rGyr and SASA values and the higher the MolSA and PSA values, the better the stability of the generated complex [31]. In accordance with the MD simulation results, the complex A6R–Fludarabine exhibited the lowest values of rGyr (3.4 Å) and SASA (125 Å) and the highest PSA and MolSA values compared to the complexes of Fludarabine with D8L and F13L.

**Figure 6:**
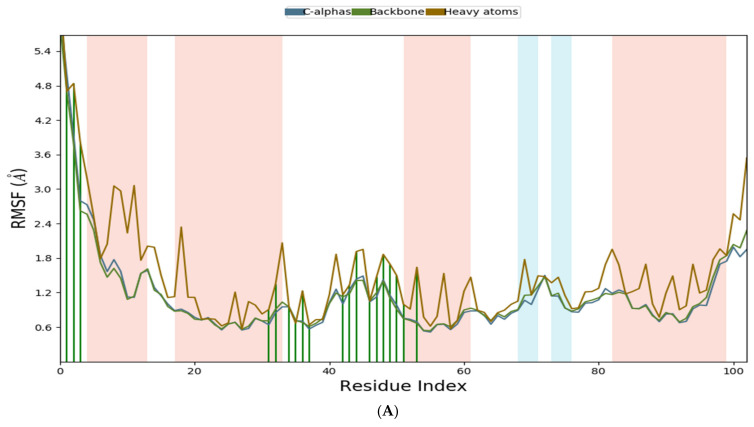

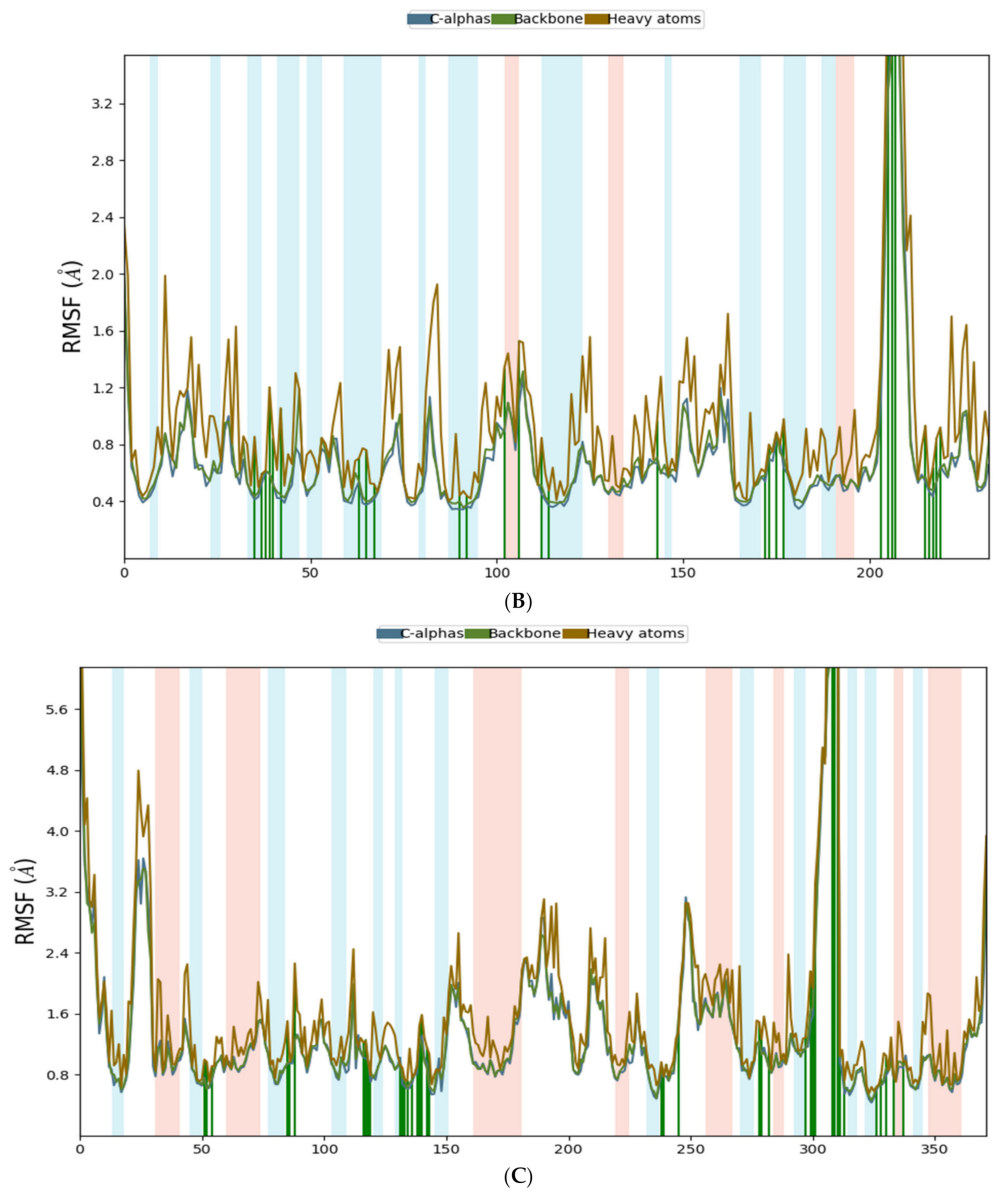
Time-dependent RMSF plot of protein backbones, C-alpha, and heavy atoms during the interaction with the ligand (horizontal green lines). Numbers on the x-axis indicate the number of protein residues, while the y-axis indicates the protein fluctuation in Å. (**A**). DNA-Dependent RNA Polymerase subunit (A6R) and Fludarabine, (**B**). Carbonic anhydrase (GAG-binding IMV membrane protein) (D8L) and Fludarabine, (**C**). Palmitoylated extracellular enveloped virus (EEV) membrane glycoprotein (F13L) and Fludarabine.

**Figure 7:**
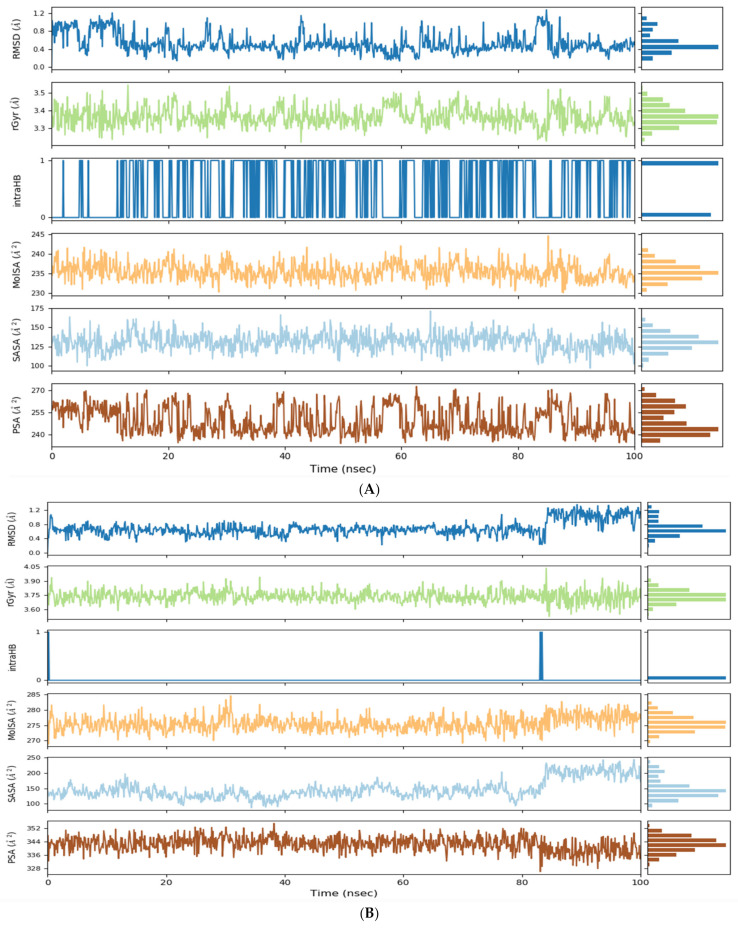

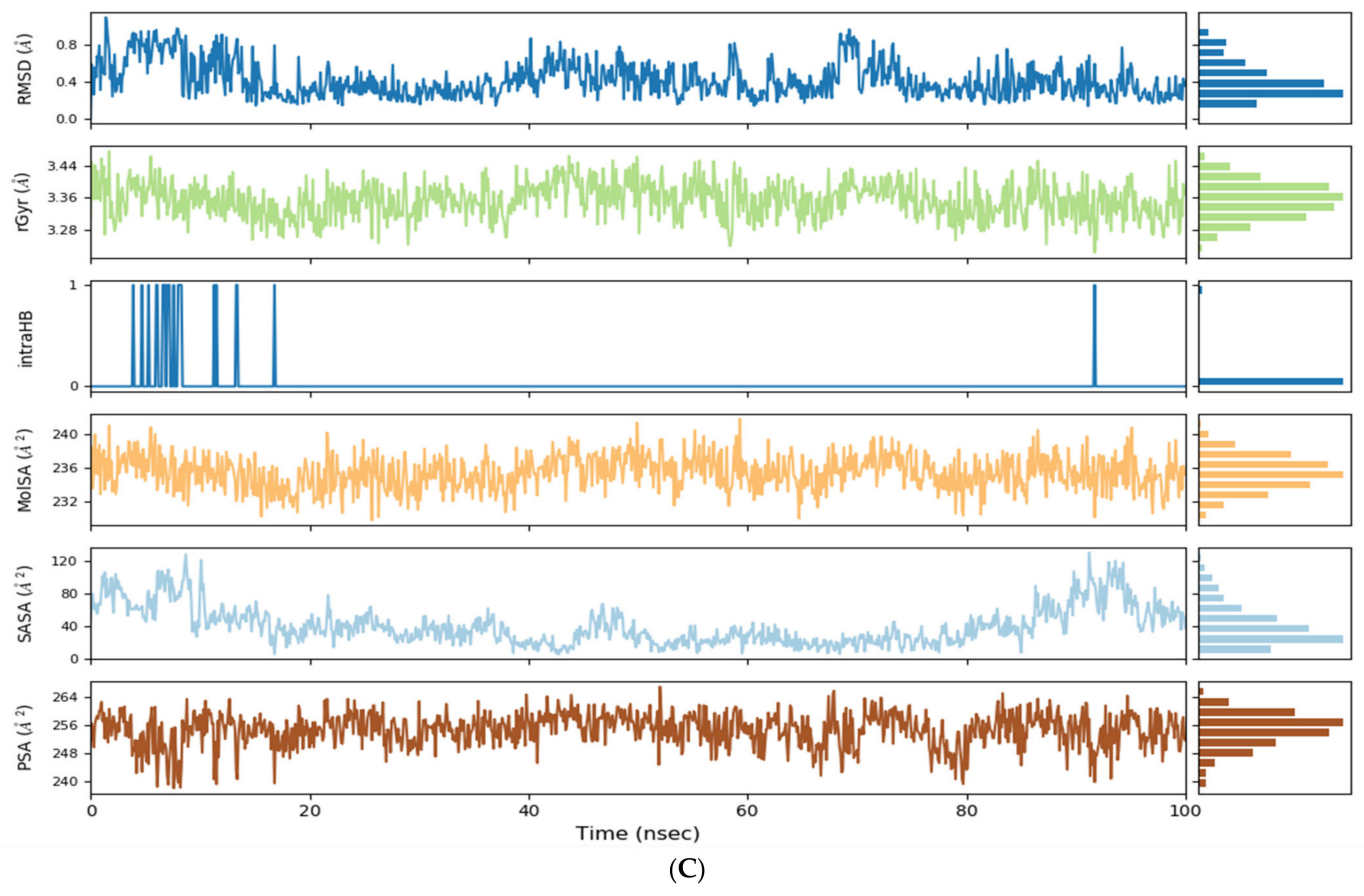
Fludarabine RMSD, rGyr, intramolecular H-bond (intraHB), molecular surface area (MolSA), solvent-accessible surface area (SASA), polar surface area (PSA) calculated during the MD simulation (100 ns). (**A**). A6R, (**B**). D8L, (**C**). F13L.

### 2.5. MM/GBSA Analysis

MM/GBSA is used to estimate the relative binding affinities of ligands to a protein active site. The results obtained indicated the approximate free energy generated by the binding, with more negative values indicating stronger binding affinities [32]. As shown in Table 2, the complex F13L–Fludarabine showed the lowest predictive binding energy of −51.65 kcal/mole, in a range of −72.88 to −24.72 kcal/mole, and a relatively high standard deviation (10.24). The measured value was better than that of the binding free energy (−44.84 kcal/mole) of the control (Tecovirimat) and F13L. The interaction of Fludarabine and A6R exhibited an average binding energy (ΔG*bind*) of −44.62 kcal/mole, in a range between −53.26 and −35.49, and a low value of standard deviation (3.76), which indicated the stability of the ligand during the whole simulation time. This result is consistent with the MD simulation results. The complex of D8L–Fludarabine exhibited an average binding energy of −39.47 kcal/mole, in the range between −55.62 and −26.06, indicating a lower stability compared to that of the previous complexes. This finding is reflected in the high deviation of the ligand from the protein backbones during the MD simulation process (Table 2).

### 2.6. Absorption, Distribution, Metabolism, Excretion, and Toxicity (ADME/T) Profiles

Similar to the control antiviral agents (Cidofovir and Tecovirimat), Fludarabine showed good pharmacokinetic properties based on computed ADME parameters. No inhibitory activity was reported toward all investigated cytochrome P450 enzymes (CYP_2C19, CYP_2C9, CYP_2D6, CYP_2D6, CYP_3A4, and CYP_3A4). Different toxicity parameters were also measured using tests such as the Ames test, the mouse and rat cell carcinogenicity test, and the hERG (human ether-à-go-go-related gene) inhibition test, revealing normal values appropriate for humans. The reference compounds showed a mild carcinogenic effect in mice and rats. Additionally, Fludarabine showed a 50% Human Intestinal Absorption (HIA) value better than that of Cidofovir (12%) and lower than that of Tecovirimat (96%). The lower values of Tecovirimat toxicity are in agreement with previous in vitro studies [17]. Fludarabine showed no mutagenic effect in the Ames test, which agrees with a previous report [33]. The predicted ADME/T properties are presented in Table S1.

## 3. Methods

### 3.1. Homology Modeling and Multiple Sequence Alignment

Three essential proteins of MPXV were selected in this study; they were obtained from the recently emerged Monkeypox virus isolate (MPXV_USA_2022_MA001) identified from a confirmed case in Massachusetts, United States, May 2022 [9]; the protein sequences were obtained from GenBank (ON563414.3). Three essential proteins of MPXV were targeted in this study, i.e., the essential protein for viral replication DNA-Dependent RNA Polymerase subunit (RPO19) (A6R) (URK20553.1), an important protein in viral cell entry carbonic anhydrase (GAG-binding IMV membrane protein) (D8L) (URK20542.1), and the palmitoylated extracellular enveloped virus (EEV) membrane glycoprotein (encoded by F13L gene) (URK20480.1) which is required for the catalysis of the envelopment of intracellular mature virus particles [1,15].

The A6R, D8L, and F13L proteins of the newly identified MPXV were obtained from the recently released complete genome sequences (ON563414.3) submitted to GenBank. They were then aligned to different poxviruses proteins to identify the conserved domains of the selected proteins. Multiple sequence alignment was performed using BioEdit 7.0.9 software [34].

The three-dimensional (3D) structure of the proteins was generated using the homology modeling approach. The Prime tool of the Maestro interface (Schrödinger Release 2020-3) was utilized for model prediction [35]. The structure prediction wizard in the Prime interface was used for model generation, the Prime BLAST homology search was used for the identification of the most similar template in the PDB database, and then the templates with the best score were selected for model generation.

The crystal structure of the vaccinia DNA-dependent RNA polymerase (PDB ID: 6RFL_C) was used as a template for the generation of the MPXV A6R protein model, which showed 97% identity and positivity. The crystal structure of the ectodomain of the vaccinia virus envelope protein D8L (PDB ID: 4E9O_A) was used as a template for the MPXV D8L protein, which showed 93% identity and 96% positivity. The crystal structure of *Serratia plymuthica* phospholipase D (PDB ID: 7E0M_A) was used as a template for F13L, which showed 20% identity and 43% positivity. Proteins with high amino acid sequence similarity (A6R and D8L) were aligned with the Prime method; then, models were generated from the aligned sequence using the knowledge-based method, while the protein with low sequence similarity was aligned with the Prime STA method, and the model was generated with the energy-based method. Loops that contained gaps in the aligned sequences were refined by the Prime loops refinement tool. The Maestro Ramachandran plot was used to estimate the quality of the built structures.

### 3.2. Active Site Prediction

Prior to the molecular docking study, the binding pocket of each protein was identified using the Maestro Interface SiteMap tool [36]. The best binding pocket was then determined according to the druggability score (Dscore), SiteScore, and results of a previous publication [23]. Pockets with a DScore value of more than or equal to 0.1 were considered very druggable binding sites, the binding sites with a DScore = 0.8–1.0 were considered druggable, binding sites with a Dscore = 0.7–0.8 were considered intermediately druggable, and the sites with a Dscore less than or equal 0.7 were considered to have difficult druggability [26].

### 3.3. Structure Preparation and Molecular Docking

The proteins were prepared for molecular docking via the Protein Preparation Wizard in the Maestro interface [37]. The proteins were subjected to a preprocess step which includesdthe addition of hydrogen bonds, the creation of disulfide bonds, and the generation of hetero states using Epik at pH 7.0. The protein energy was then minimized via the OPLS3e force field. Over 1600 FDA-approved compounds were obtained from the ZINC 15 database, and the compounds structures were obtained in sdf format and then prepared using Maestro LigPrep [38]. The ligands ionization state was generated at pH 7.0 (±2.0) using the Epik ionizer.

The binding affinity of the FDA-approved compounds toward the protein active sites was screened via the High-Throughput Virtual Screening (HTVS) module in the Maestro Ligand Docking tool. The top 200 compounds were re-docked using standard docking (SP), then the top 100 compounds were re-docked again using extra precision (XP) docking. During the docking process, the ligands were set flexible, while the proteins were rigid. Due to the adoption of Epik for ligands preparation, Epik state penalties were added during the docking process to obtain higher-energy states.

The docking results were compared to those of Tecovirimat (ST-246 or TPOXX^®®^), which was used as the F13L inhibitor, and of chondroitin sulfate, which mediates the binding of D8L to the host cell and facilitates the adsorption of intracellular mature virions [27]. Cidofovir was used as a control for A6R, due to its reported in vitro activity on DNA polymerase of MPXV [39]. The docking complexes were visualized using Discovery studio [40].

### 3.4. Molecular Dynamic (MD) Simulation

The MD simulation was conducted to study the stability and interactions of the docked complexes. The whole process of MD simulation was conducted on Maestro Desmond Module (Schrodinger Release 2020-3). Before the running of the MD simulation, the energy of the entire system was first minimized using the OPLS3e force field. The complexes of Fludarabine and the three selected proteins were selected for the MD simulation, because Fludarabine exhibited multiple activities on all of the selected proteins, showing the best XP docking score with A6R and good docking scores with D8L and F13L. The complexes were soaked in a 10 Å orthorhombic box to provide a TIP4P water solvent model, then the system was neutralized by the addition of the required number of Na^+^ and Cl^−^ ions. Finally, the system was equilibrated at 300 K and a pressure (bar) of 1.01 and run for 100 ns. The generated simulation trajectories were analyzed by the simulation interaction diagram (SID) of the Desmond module. We also used SID to analyze the complex stability by estimating the Radius of gyration (rGyr), intramolecular hydrogen bonds (HBs), molecular surface area (MolSA) (equivalent to a van der Waals surface area), solvent accessible surface area (SASA), and polar surface area (PSA) [31].

### 3.5. MM-GBSA

Molecular Mechanics Generalized Born Surface Area (MM-GBSA) is an important parameter used to assess docking poses, binding affinity, and structural stability [41]. MM-GBSA is a method that uses generalized Born models for the estimation of the binding free energy of a small molecule with a protein [42].

For the calculation of the binding free energy (ΔG_bind), we used the Schrodinger thermal_mmgbsa.py script. All trajectories (1000) generated from the MD simulation were used as input, with a step size of 10 [43,44]. The following MM-GBSA methodology was used

ΔG_bind_ = G_complex_ − G_receptor_ − G_ligand_

where G_complex_ represents the free energy of the complex, G_receptor_ represents the receptor energy, and G_ligand_ represents the ligand-free energy [41].

### 3.6. Absorption, Distribution, Metabolism, Excretion, and Toxicity (ADME/T) Profiles

In silico prediction of ADME/T was achieved via the PreADMET web server and admetSAR. We estimated the absorption, distribution, metabolism, excretion, and toxicity of Fludarabine and the control compounds (Cidofovir and Tecovirimat). The rules of five were used to estimate the drug-likeness of the compounds [31]; ADME was investigated via the analysis of cytochrome P450 (CYP) inhibition, blood–brain barrier (BBB) permeability, binding of plasma proteins, skin permeability, human intestinal absorption (HIA), and P-glycoprotein (Pgp) inhibition. Compounds’ toxicity was investigated via hERG inhibition, a mouse and rat carcinogenicity test, and the Ames test [31].

## 4. Conclusions

Monkeypox is a zoonotic contagious disease that has recently re-emerged in different countries worldwide. Unfortunately, there is no available treatment for MPXV infection [22]. In the present study, we utilized different computational methods, including computational modeling, molecular docking, MD simulation, and MM-GBSA, to identify potential inhibitors of MPXV essential proteins. In this study, we identified an FDA-approved drug (Fludarabine) that has potential inhibitory activity on different MPXV essential proteins. Fludarabine, which is used as an anticancer drug, showed the best in-silico activity on the essential protein for viral replication termed DNA-Dependent RNA Polymerase (A6R). It also showed good activity on a protein important for viral cell entry (D8L) and on the F13L protein, which is required for the catalysis of the envelopment of intracellular mature virus particles. ADMET profiling showed that the compound has low toxicity and good pharmacokinetic properties similar to those of the other antiviral agents (Tecovirimat and Cidofovir) used as a control in this study. On the basis of the results of this study, we recommend more in vitro and in vivo studies on this compound as a starting point to develop a novel treatment against MPXV.

## Figures and Tables

**Table 1:** Extra-precision (XP) docking score of the best compounds.

Compound Name	ZINC_ID	Pharmacological Function	XP GScore	Glide Energy
**A6R**				
Fludarabine	ZINC000004216238	Anti-cancer	−7.53	−30.234
Adenosine	ZINC000002169830	Antidysrhythmics	−7.448	−33.69
Adrenor	ZINC000000057624	Anti-hypotensive	−6.408	−33.238
Cladribine	ZINC000003798064	Anti-cancer	−6.121	−34.906
Azacitidine	ZINC000003861768	Anti-cancer	−6.435	−32.87
Epinephrine	ZINC000000039089	Hormone and neurotransmitter	−6.011	−32.531
Epivir	ZINC000000012346	Antiviral	−5.903	−29.345
Cytarabine	ZINC000003795098	Anti-cancer	−5.89	−31.304
Zolmitriptan	ZINC000000015515	Used to treat the symptoms of migraine	−5.656	−34.463
Levonordefrin	ZINC000000034157	Vasoconstrictor	−5.616	−27.364
Cidofovir	ZINC000001530600		−4.5	−30.9
**D8L**				
Iohexol	ZINC000003830943	Diagnostic contrast agent	−9.124	−51.614
Iopromide	ZINC000003830957	Low-osmolar, non-ionic contrast agent	−9.054	−58.806
Isovue-M	ZINC000003830947	Diagnostic contrast agent	−7.56	−54.575
Risedronate	ZINC000001531009	Use to treat osteoporosis	−7.736	−41.476
Ioxilan	ZINC000085540219	Diagnostic contrast agent	−7.381	−48.531
Risedronate	ZINC000001531009	Use for slowing bone loss	−7.082	−39.754
Fludarabine	ZINC000003927870	Anti-cancer	−6.642	−42.27
Pitavastatin	ZINC000001534965	Used to lower LDL (bad) cholesterol	−6.147	−40.438
Chondroitin sulfate	ZINC000012494114		−9.124	−61.519
**F13L**				
Ioxilan	ZINC000085540215	Diagnostic contrast agent	−9.227	−58.068
Iohexol	ZINC000003830945	Diagnostic contrast agent	−9.029	−54.74
Iopromide	ZINC000003830957	Low osmolar, non-ionic contrast agent	−8.246	−55.739
Adenosine	ZINC000002169830	Antidysrhythmics	−8.008	−37.689
Cedax	ZINC000003871967	Antibacterial	−7.985	−43.492
Idarubicin	ZINC000003920266	Anti-cancer	−7.984	−41.742
Dobutamine	ZINC000000057278	Used in the treatment of cardiogenic shock	−7.769	−38.923
Fludarabine	ZINC000003927870	Anti-cancer	−7.66	−40.897
*Tafluprost*	ZINC000013912394	Used to treat glaucoma	−7.452	−39.127
Zetia	ZINC000003810860	Used to treat high cholesterol levels in adults	−7.406	−42.326
Tecovirimat	ZIN0000C35323125		−9.22	−45.100

**Table 2:** Binding affinities and ligand properties of the best active compounds against MPXV proteins (A6R, D8L, and F13L).

Complex.	MM/GBSA			rGyr (Å)	MolSA (Å)	SASA (Å)	PSA (Å)
	dG(NS) Average	Range	Standard Deviation				
A6R–Fludarabine	−44.62	−53.26 to −35.49	3.76	3.4	236	125	260
D8L–Fludarabine	−39.47	−55.62 to −26.06	6.23	4.32	448	300	340
F13L–Fludarabine	−51.65	−72.88 to −24.72	10.24	4.1	420	160	310
A6R–Cidofovir	−16.15	−36.9 to 0.708	12.02	3.28	240	380	290
F13L–Tecovirimat	−44.84	−53.17 to −28.73	4.04	4.7	316	120	117
D8L–Chondroitin sulfate	−54.41	−73.42 to −34.11	8.58	4.05	348	140	440

## Data Availability

All data are included in the manuscript and the additional files.

## Supplementary Materials

The following supporting information can be downloaded at: https://www.mdpi.com/article/10.3390/ph15091129/s1, Figure S1. Multiple sequence alignment of the newly emerged MPXV proteins compared to different poxviruses; Figure S2. RMSD plot (A) and histogram (B) generated from the interaction of A6R and Cidofovir during 100ns simulation; Figure S3. RMSD plot (A) and histogram (B) generated from the interaction of D8L and Chondroitin sulfate during 100ns simulation; Figure S4. RMSD plot (A) and histogram (B) generated from the interaction of F13L and Tecovirimat during 100ns simulation; Table S1. In silico ADME/T profiling of Fludarabine and the control compounds (Cidofovir, and Tecovirimat).
