# Supplementary material for: Fludarabine, a Potential DNA-Dependent RNA Polymerase Inhibitor, as a Prospective Drug against Monkeypox Virus: A Computational Approach
Source: Pharmaceuticals (Basel). 2022 Sep 9;15(9):1129. doi: 10.3390/ph15091129 (PMC9504824; doi:10.3390/ph15091129)
Supplement: Supplementary file 1 [file pharmaceuticals-15-01129-s001.zip › pharmaceuticals-1865657-supplementary.pdf]

A

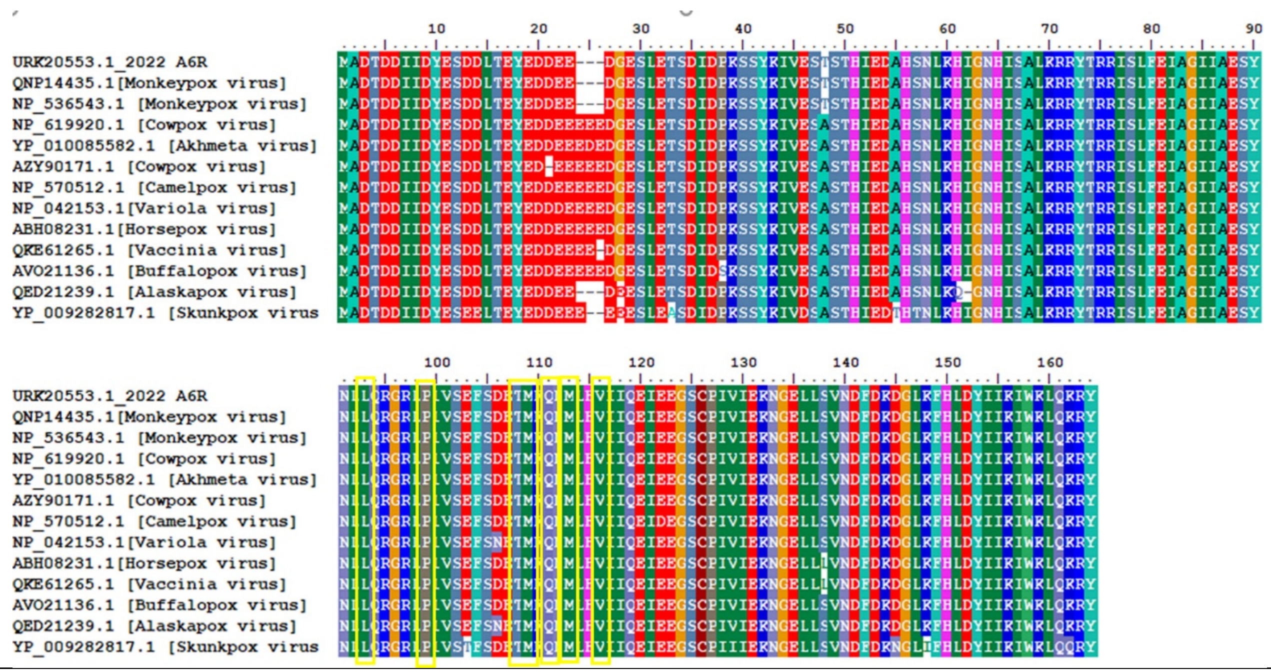

10 20 30 40 50 60 70 80 90
URK20553.1_2022 A6R
QNP14435.1[Monkeypox virus]
NP_536543.1 [Monkeypox virus]
NP_619920.1 [Cowpox virus]
YP_010085582.1 [Akhmeta virus]
AZY90171.1 [Cowpox virus]
NP_570512.1 [Camelpox virus]
NP_042153.1[Variola virus]
ABH08231.1[Horsepox virus]
QKE61265.1 [Vaccinia virus]
AVO21136.1 [Buffalopox virus]
QED21239.1 [Alaskapox virus]
YP_009282817.1 [Skunkpox virus
100 110 120 130 140 150 160
URK20553.1_2022 A6R
QNP14435.1[Monkeypox virus]
NP_536543.1 [Monkeypox virus]
NP_619920.1 [Cowpox virus]
YP_010085582.1 [Akhmeta virus]
AZY90171.1 [Cowpox virus]
NP_570512.1 [Camelpox virus]
NP_042153.1[Variola virus]
ABH08231.1[Horsepox virus]
QKE61265.1 [Vaccinia virus]
AVO21136.1 [Buffalopox virus]
QED21239.1 [Alaskapox virus]
YP_009282817.1 [Skunkpox virus

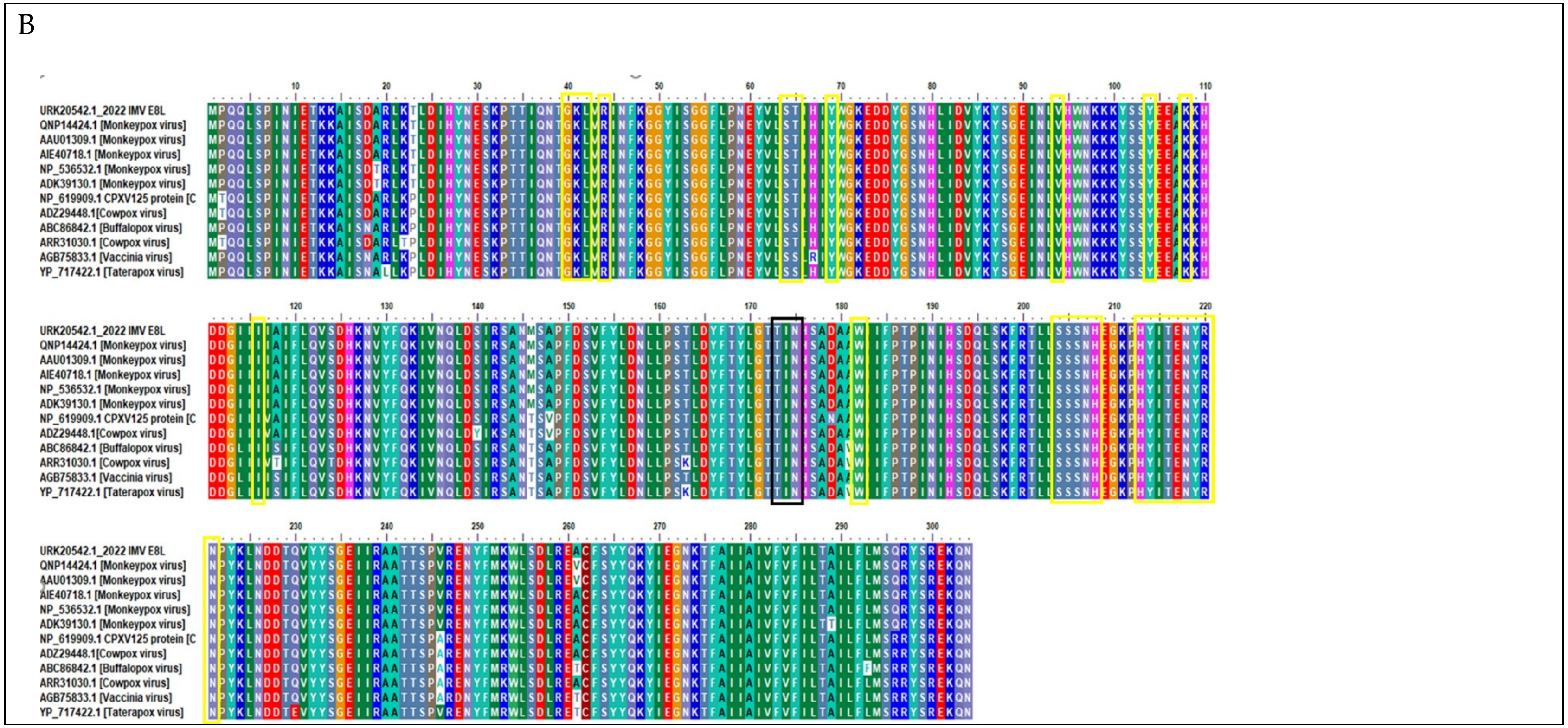
B
URK20542.1_2022 IMV E8L
QNP14424.1 [Monkeypox virus]
AAU01309.1 [Monkeypox virus]
AIE40718.1 [Monkeypox virus]
NP_536532.1 [Monkeypox virus]
ADK39130.1 [Monkeypox virus]
NP_619909.1 CPXV125 protein [C
ADZ29448.1[Cowpox virus]
ABC86842.1 [Buffalopox virus]
ARR31030.1 [Cowpox virus]
AGB75833.1 [Vaccinia virus]
YP_717422.1 [Taterapox virus]

C

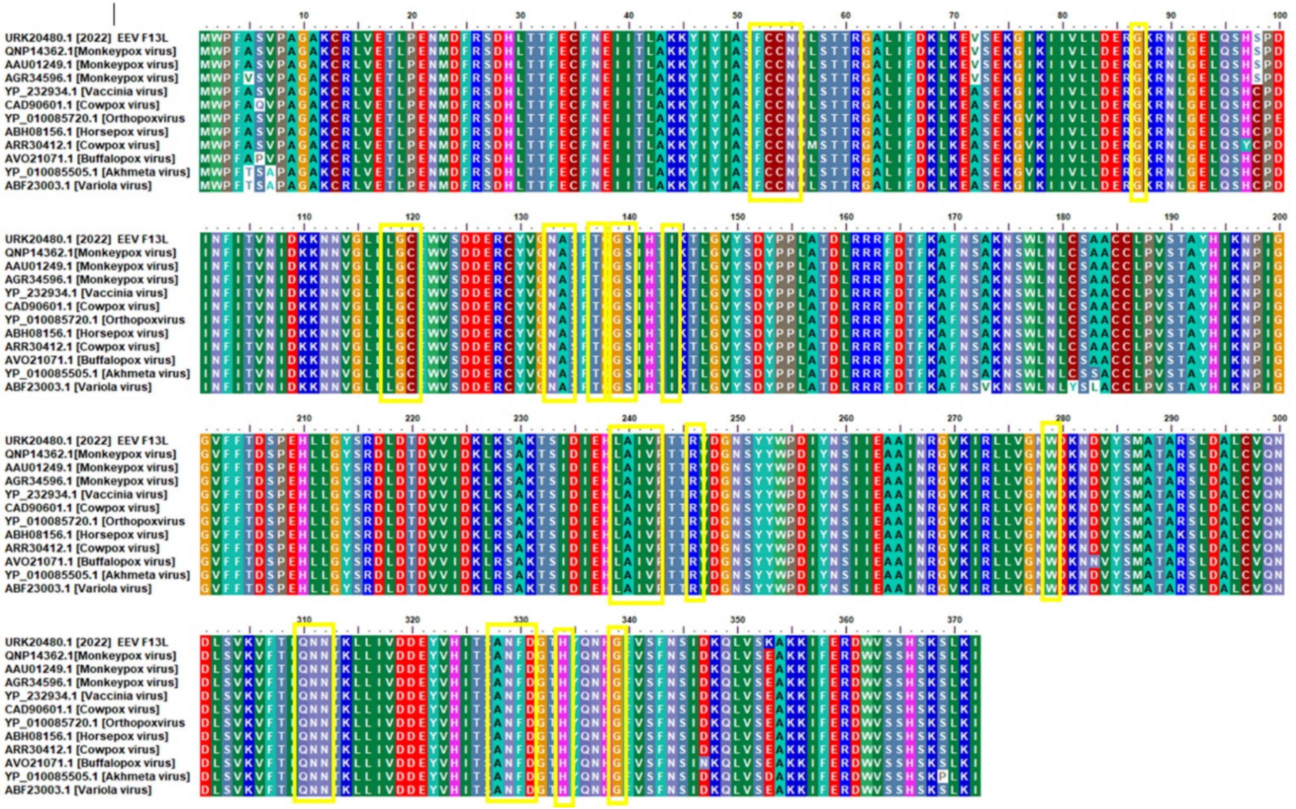

Figure S1. Multiple sequence alignment of the newly emerged MPXV proteins (in the first line) compared to different poxviruses, the conserved binding site residues are shown in yellow horizontal blocks. A. RNA polymerase subunit (A6R), B. The carbonic anhydrase (GAG-binding IMV membrane protein) (D8L), C. The palmitylated extracellular enveloped virus (EEV) membrane glycoprotein (F13L).

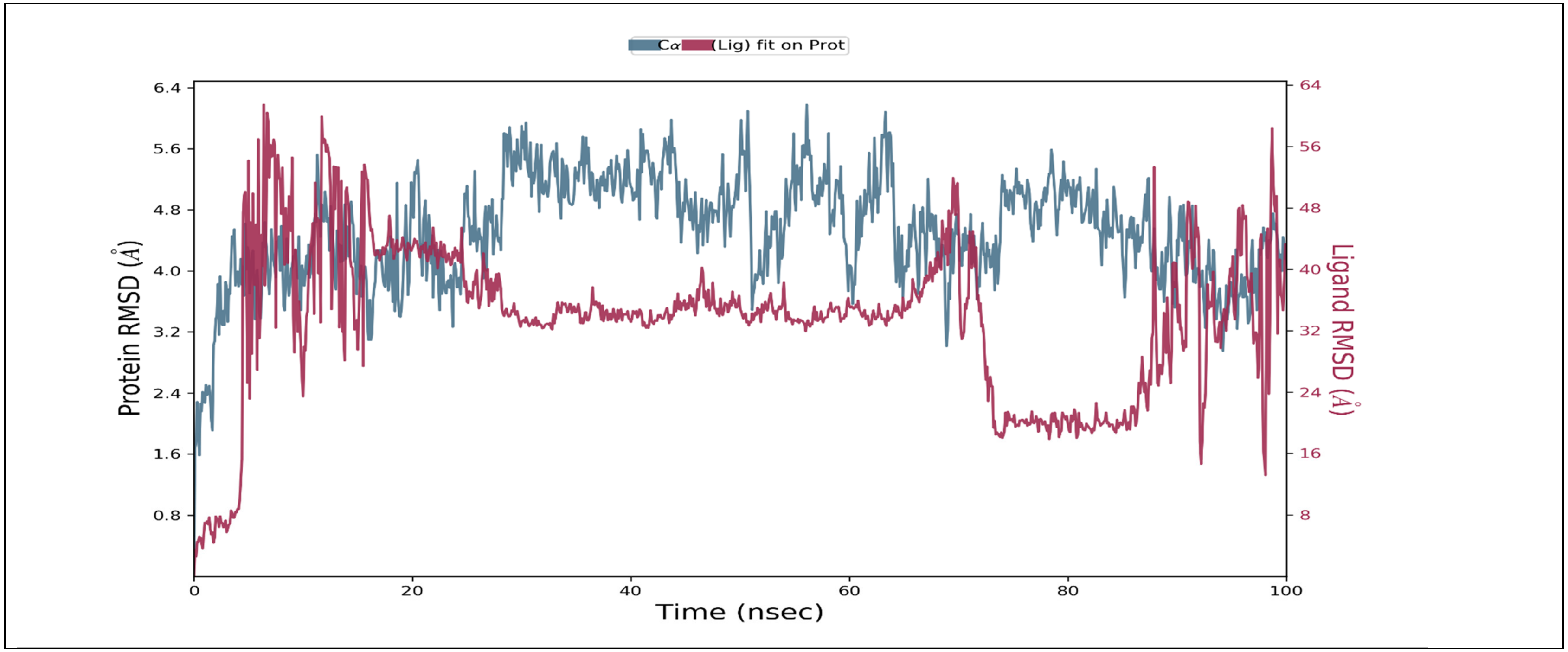

Cα
(Lig) fit on Prot
Protein RMSD (Å)
6.4
5.6
4.8
4.0
3.2
2.4
1.6
0.8
64
56
48
40
32
24
16
8
Ligand RMSD (Å)
0
20
40
60
80
100
Time (nsec)

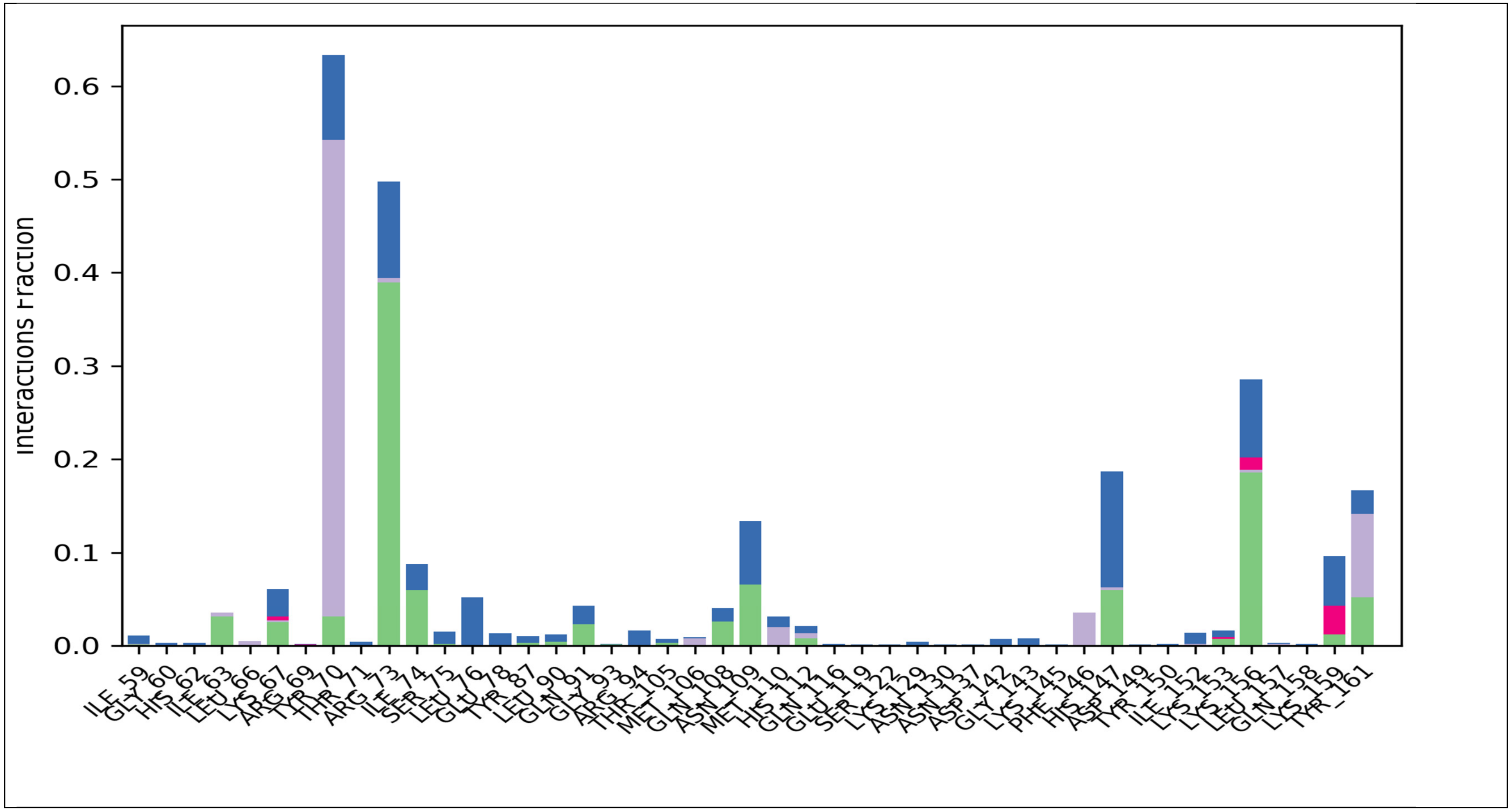

Figure S2. RMSD plot (A) and histogram (B) generated from the interaction of A6R and Cidofovir during 100ns simulation. The RMSD plot shows ligands in red and protein C-alpha atoms in blue, the histogram shows H-bonds (green), water bridges (blue), and hydrophobic (purple).

A

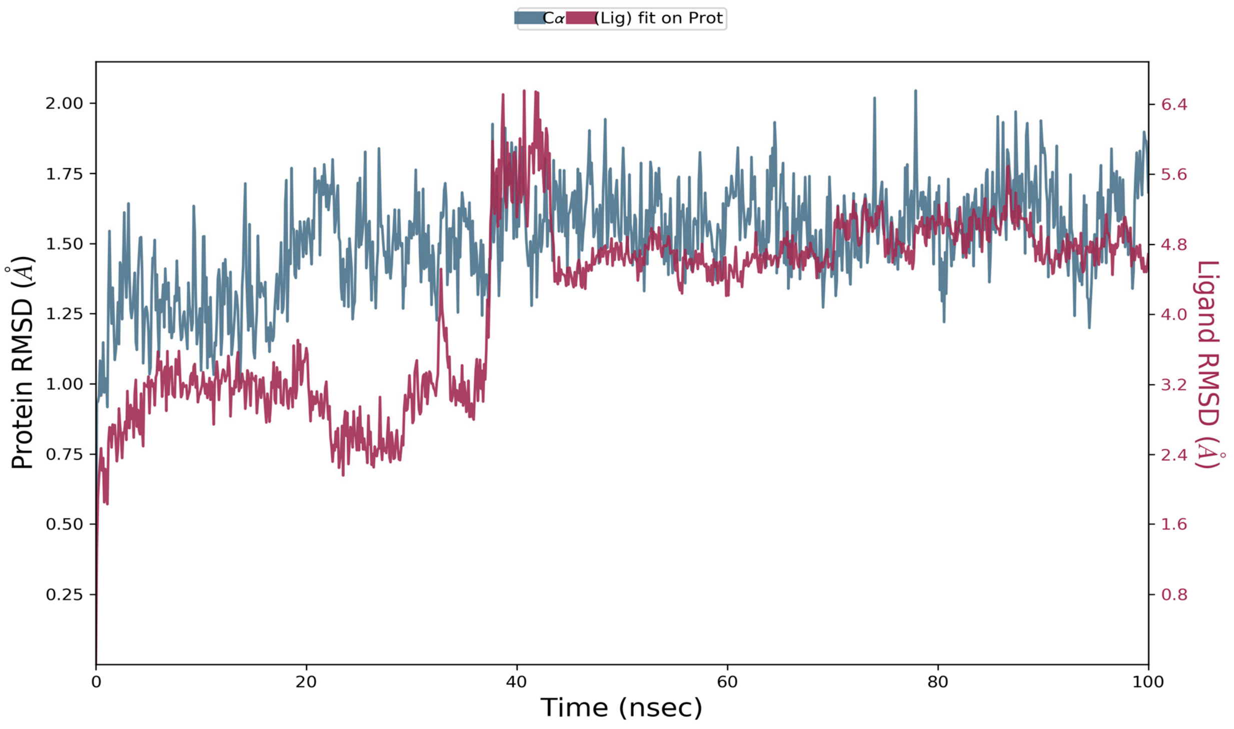

Cα
(Lig) fit on Prot
Protein RMSD (Å)
Ligand RMSD (Å)
Time (nsec)
2.00
1.75
1.50
1.25
1.00
0.75
0.50
0.25
6.4
5.6
4.8
4.0
3.2
2.4
1.6
0.8
0
20
40
60
80
100

B

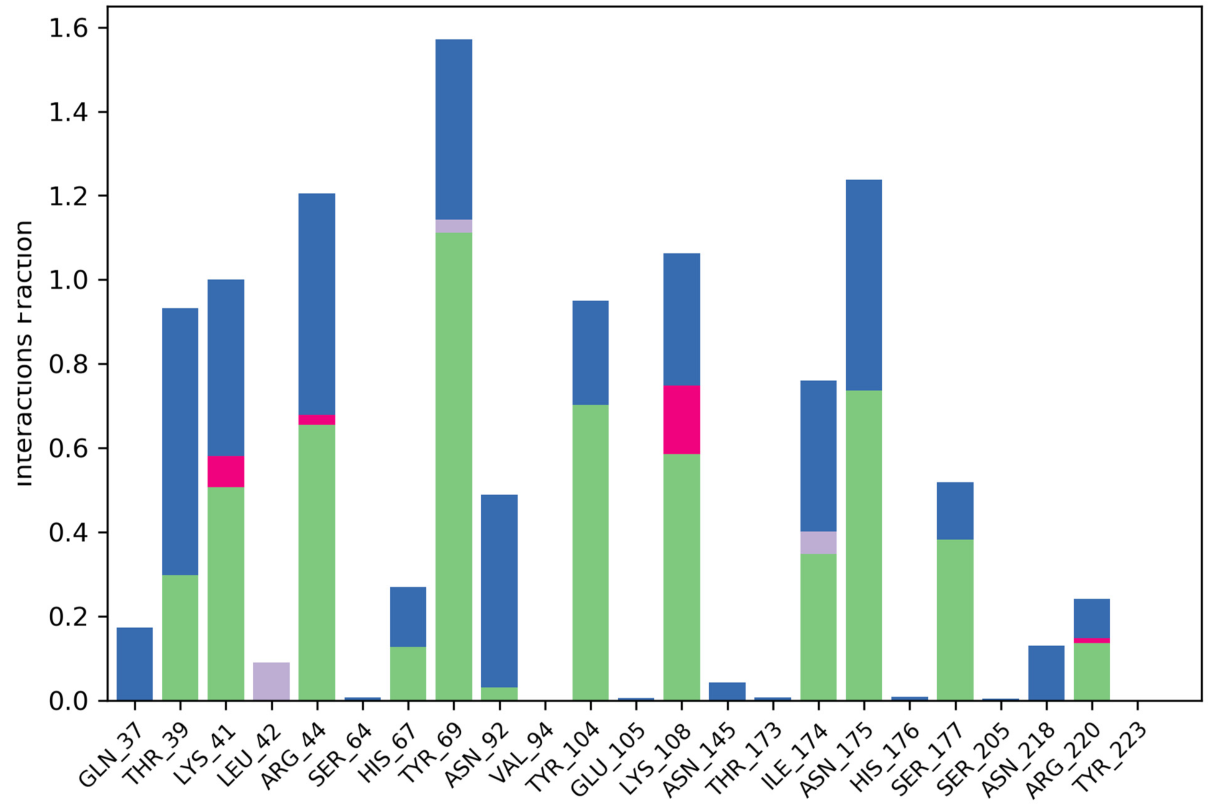

Figure S3. RMSD plot (A) and histogram (B) generated from the interaction of D8L and Chondroitin sulfate during 100ns simulation. The RMSD plot shows ligands in red and protein C-alpha atoms in blue, the histogram shows H-bonds (green), water bridges (blue), and hydrophobic (purple).

A

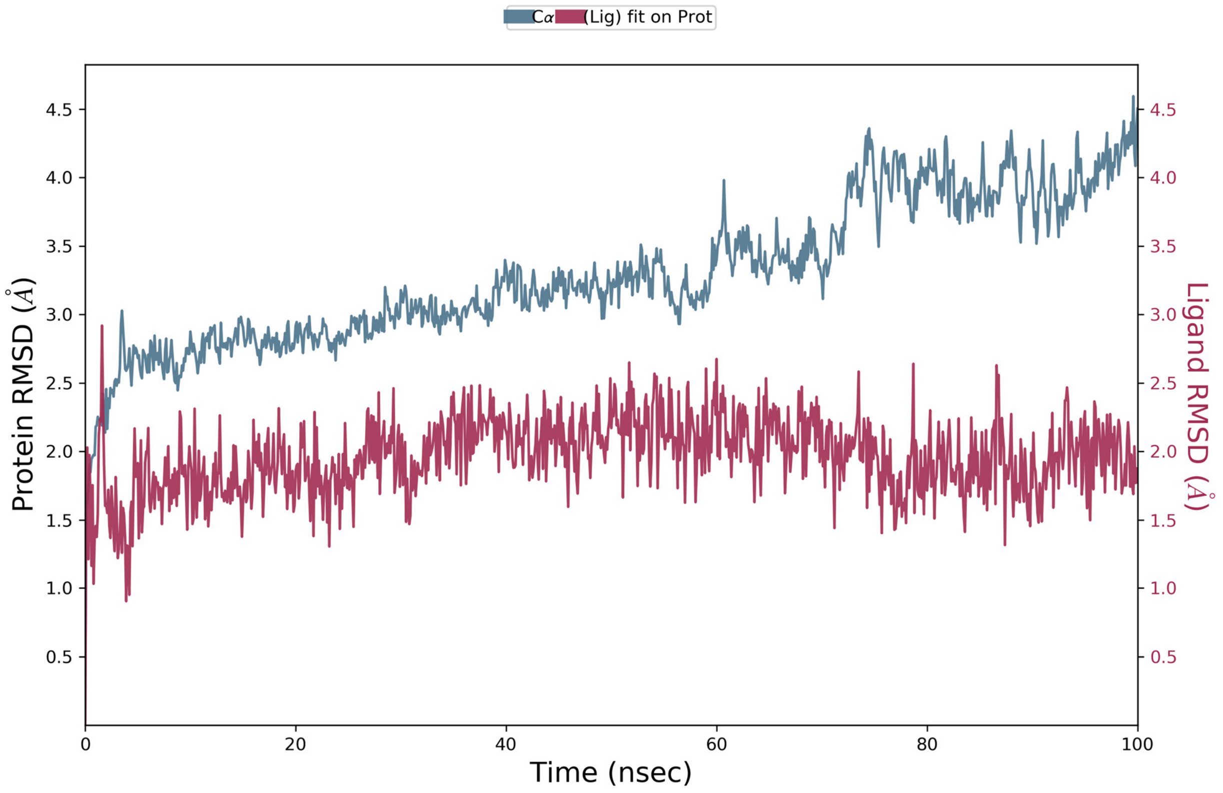

Cα
(Lig) fit on Prot
Protein RMSD (Å)
Ligand RMSD (Å)
Time (nsec)
0
20
40
60
80
100
0.5
1.0
1.5
2.0
2.5
3.0
3.5
4.0
4.5

B

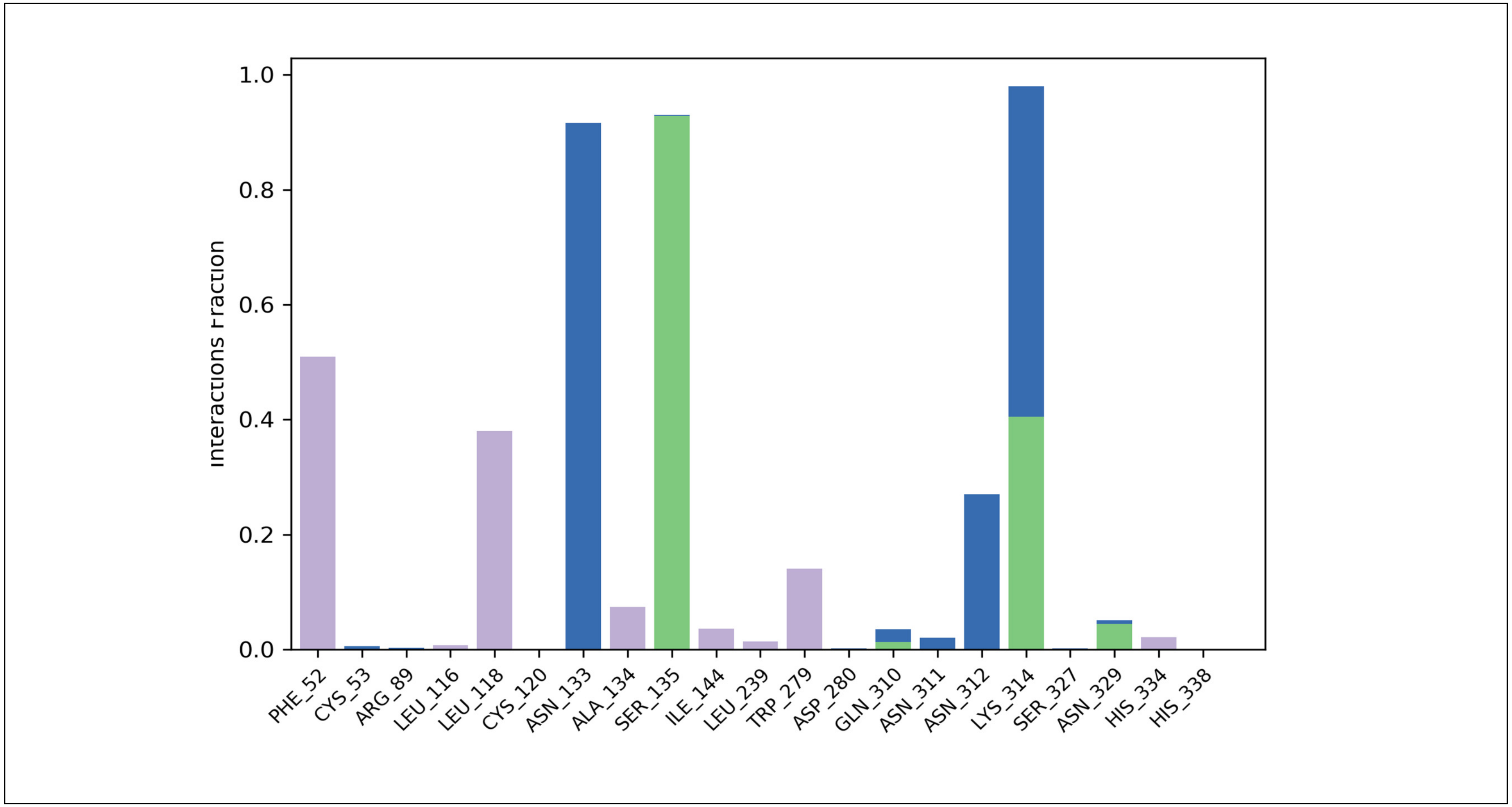

Figure S4. RMSD plot (A) and histogram (B) generated from the interaction of F13L and Tecovirimat during 100ns simulation. The RMSD plot shows ligands in red and protein C-alpha atoms in blue, the histogram shows H-bonds (green), water bridges (blue), and hydrophobic (purple).

Table S1. In silico ADME/T profiling of Fludarabine and the control compounds (Cidofovir, and Tecovirimat)

		**Fludarabine**	**Cidofovir**	**Tecovirimat**
**Druglikeness**	**ID**	**Value**	**Value**	**Value**
	CMC_like_Rule	Not qualified	Not qualified	Qualified
	CMC_like_Rule_Violation_Fields	AlopP98_value	AlopP98_value	
	CMC_like_Rule_Violations	1	1	0
	Lead-like_Rule_Violation_Fields	AlopP98_value	AlopP98_value	Molecular_weight
	Lead_like_Rule	Violated	Violated	Violated
	Lead_like_Rule_Violations	1	1	1
	MDDR_like_Rule	Mid-structure	Mid-structure	Mid-structure
	MDDR_like_Rule_Violation_Fields	No_Rotatable_bonds	No_Rings, No_Rotatable_bonds	No_Rotatable_bonds
	MDDR_like_Rule_Violations	1	2	1
	Rule_of_Five	Suitable	Suitable	Suitable
	Rule_of_Five_Violation_Fields			
	Rule_of_Five_Violations	0	0	0
	WDI_like_Rule	In 90% cutoff	In 90% cutoff	In 90% cutoff
	WDI_like_Rule_Violation_Fields			
	WDI_like_Rule_Violations	0	0	0
**ADMET**	**ID**	**Value**	**Value**	**Value**
	AlogP98_value	-1.4514	-2.9031	2.0184
	AMolRef	64.0592	59.7426	88.9481
	BBB	0.17172	0.041102	0.038807
	Buffer_solubility_mg_L	6473.68	193058**	2266.85
	Caco2	17.9654	12.4524	21.1291
	CYP_2C19_inhibition	Non	Non	Non
	CYP_2C9_inhibition	Non	Non	Non
	CYP_2D6_inhibition	Non	Non	Non
	CYP_2D6_substrate	Non	Non	Non

	CYP_3A4_inhibition	Non	Non	Non
	CYP_3A4_substrate	Non	Non	Non
	HIA	49.22237	12.0661	95.94375
	MDCK	0.587766	2.39335	0.513086
	Pgp_inhibition	Non	Non	Non
	Plasma_Protein_Binding	13.90559	61.46186	83.60271
	Pure_water_solubility_mg_L	4976.13	2.20E+06	14.4512
	Skin_Permeability	-5.19154	-4.84222	-3.30246
	Solvation_Free_Energy	-22.080000**	-29.920000**	-19.420000**
**Toxicity**	**ID**	**Value**	**Value**	**Value**
	algae_at	0.23856	0.582289	0.072835
	Ames_test	Non AMES toxic	Non AMES toxic	Not predicted
	Carcino_Mouse	negative	positive	positive
	Carcino_Rat	negative	positive	positive
	daphnia_at	4.2423	32.2172	0.110165
	hERG_inhibition	low_risk	low_risk	medium_risk
	medaka_at	23.2786	1029.05	0.021295
	minnow_at	12.2754	148.286	0.039285
	TA100_10RLI	negative	negative	negative
	TA100_NA	positive	negative	negative
	TA1535_10RLI	positive	positive	negative
	TA1535_NA	negative	negative	negative
